# Is there a relationship between two different anesthetic methods and postoperative length of stay during radical resection of malignant esophageal tumors in China?: a retrospective cohort study

**DOI:** 10.1186/s12871-022-01775-6

**Published:** 2022-07-25

**Authors:** Jieping Yang, Xukeng Guo, Zonggui Zheng, Weiqi Ke

**Affiliations:** 1 Department of Anesthesiology, The First Affiliated Hospital of Shantou University Medical College, No. 57 Changping Road, Jinping District, Shantou City, Guangdong Province China; 2 Department of Anesthesiology, The Third People’ Hospital of Shantou, No. 12 Haipang Road, Haojiang District, Shantou City, Guangdong Province China

**Keywords:** General anesthesia, Epidural anesthesia, Postoperative length of stay, Esophageal cancer

## Abstract

**Background:**

Data providing a relationship between the anesthetic method and postoperative length of stay (PLOS) is limited. We aimed to investigate whether general anesthesia alone or combined with epidural anesthesia might affect perioperative risk factors and PLOS for patients undergoing radical resection of malignant esophageal tumors.

**Methods:**

The study retrospectively analyzed the clinical data of 680 patients who underwent a radical esophageal malignant tumor resection in a Chinese hospital from January 01, 2010, to December 31, 2020. The primary outcome measure was PLOS, and the secondary outcome was perioperative risk-related parameters that affect PLOS. The independent variable was the type of anesthesia: general anesthesia (GA) or combined epidural-general anesthesia (E-GA). The dependent variable was PLOS. We conducted univariate and multivariate logistic regression and propensity score matching to compare the relationships of GA and E-GA with PLOS and identify the perioperative risk factors for PLOS. In this cohort study, the confounders included sociodemographic data, preoperative chemotherapy, coexisting diseases, laboratory parameters, intraoperative variables, and postoperative complications.

**Results:**

In all patients, the average PLOS was 19.85 ± 12.60 days. There was no significant difference in PLOS between the GA group and the E-GA group either before or after propensity score matching (20.01 days ± 14.90 days vs. 19.79 days ± 11.57 days, *P* = 0.094, 18.09 ± 9.71 days vs. 19.39 ± 10.75 days, *P* = 0.145). The significant risk factors for increased PLOS were lung infection (β = 3.35, 95% confidence interval (CI): 1.54–5.52), anastomotic leakage (β = 25.73, 95% CI: 22.11–29.34), and surgical site infection (β = 9.39, 95% CI: 4.10-14.68) by multivariate regression analysis. Subgroup analysis revealed a stronger association between PLOS and vasoactive drug use, blood transfusions, and open esophagectomy. The results remained essentially the same (stable and reliable) after subgroup analysis.

**Conclusions:**

Although there is no significant association between the type of anesthesia(GA or E-GA) and PLOS for patients undergoing radical esophageal malignant tumor resection, an association between PLOS and lung infection, anastomotic leakage, and surgical site infection was determined by multivariate regression analysis. A larger sample future study design may verify our results.

## Introduction

Esophageal cancer (EC) is a concerning health threat in China, ranking sixth among new cancer cases in 2020, with approximately 320,000 new claims and the 4th highest mortality rate [1, 2]. Additionally, there are areas with high EC incidence, such as the Chaoshan area of Guangdong Province [3].

The incidence of EC in China is much higher than that in Western countries, with more than half of new annual cases of EC worldwide occurring in China [2]. EC may require multiple treatment methods (more than any other solid tumor); the mainstream treatment method is radical resection of esophageal cancer, a traumatic operation with a long recovery time and a mean hospital stay of 14 days [4, 5]. Prolonged PLOS is associated with increased morbidity, mortality and resource use [5]. A longer PLOS affects the speed of recovery and increases the financial burden on patients. Previous literature and related studies have shown that optimization of anesthesia management, [6] improvement in preoperative nutritional status, [7] hospital esophagectomy volume, [8] postoperative complication-free status [9] and rapid rehabilitation after esophageal cancer resection [10, 11] may shorten PLOS.

General anesthesia (GA) and combined epidural-general anesthesia (E-GA) are two anesthesia types commonly used in radical resection of esophageal malignant tumor. However, previous findings regarding the relationship between anesthesia type and PLOS are mixed; studies either showed that anesthesia type might have an association with PLOS [12, 13] or showed no association at all [14]. This inconsistency may be due to the frequent incomparability of patient groups, a lack of transparency in selecting which patient data to include, and the reporting quality of studies comparing the effects of different anesthesia types on PLOS in patients undergoing radical resection of esophageal malignant tumor, which often lack clear reporting of all results. Thus, the representativeness and validity of these data cannot be fully determined, and therefore it remains unclear which anesthesia is the best option. Additionally, few studies have reported the association between anesthesia modality and PLOS. Therefore, we aimed to determine whether the relationship between E-GA/GA and PLOS was statistically reliable.

Specifically, we explored the relationship between the two types of anesthesia and PLOS in patients undergoing radical resection of esophageal malignant tumor in China, after adjusting for other confounders.

## Participants and methods

### Study design

In this retrospective cohort study design, we aimed to investigate whether anesthesia type (GA/E-GA) during radical esophageal malignant tumor resection has any association with PLOS. The objective, the independent variable, was anesthesia type (GA/E-GA), and the dependent variable was PLOS. PLOS was defined as the total number of days from the day of surgery to discharge. GA was general anesthesia in the form of complete intravenous anesthesia (propofol + remifentanil) or intravenous and inhalation anesthesia (sevoflurane). E-GA was based on GA combined with thoracic epidural anesthesia. Thoracic epidural anesthesia was started after a sterile preparation and insertion of an epidural catheter at the thoracic level of T6 to T8 in every patient with the same anesthesia protocol of the hospital. The study protocol includes sterile preparation with betadine, the lateral position of the patient after GA induction, insertion of Thuoy needle Gouge number 18 with the use of appropriate technique and placement of an epidural catheter, and preparation and administration of the same epidural anesthetic solution).

 Data from participants who had received radical resection of esophageal malignant tumor were obtained from the Department of Anesthesiology of the First Affiliated Hospital of Shantou University Medical College, Shantou, Guangdong Province, China. To protect patient privacy, our data did not include identifiable participant data. Data were extracted from the hospital’s electronic medical records system. The hospital’s Institutional Review Board approved the study (NO.B-2021-249). Written informed consent was waived by the Medical Ethics Committee of the First Affiliated Hospital of Shantou University School of Medicine because our study did not involve individually identifiable data or determine the treatment of patients. This study complied with the Declaration of Helsinki and adhered to the applicable STROBE guidelines.

### Variables

 We obtained the data of patients who underwent radical resection of esophageal malignant tumor from the clinical information system of the First Affiliated Hospital of Shantou University Medical College. We recorded anesthesia type as a categorical variable and divided it into GA or E-GA. The outcome variable (PLOS) was a continuous. In this study, the primary outcome measures was PLOS, and the secondary outcome was perioperative risk-related parameters that affect PLOS.

The literature lacks a precise and clinically acceptable definition of prolonged length of stay (LOS). Some studies have used the 75th percentile as a cutoff for defining prolonged LOS, although it is arbitrary [15, 16]. We defined prolonged PLOS as > 75th percentile; therefore, we considered PLOS > 21 days to indicate prolonged PLOS. We classified patient data based on this 21-day cutoff.

The medical records of eligible patients were reviewed. We included the following confounders that are perioperative risk-related parameters as listed below: (1) sociodemographic data (age, sex, and smoking status); (2) preoperative chemotherapy; (3) coexisting disease (hypertension, DM (diabetes mellitus), heart disease, or lung disease); (4) laboratory examination results (preoperative anemia, albumin, PLT (platelets), and levels of AST (aspartate transaminase), ALT (alanine transaminase), and Scr (serum creatinine)); (5) intraoperative associated variables (ASA (American Society of Anesthesiologist Physical Status), endotracheal tube type, continuous anesthesia [TIVA: total intravenous anesthesia; CIIA: combined intravenous and inhalation anesthesia], operation type [OE, open esophagectomy; MIE, minimally invasive esophagectomy], vasoactive drug use, blood transfusions, postoperative ICU admission, operation time, and blood loss); and (6) postoperative complications (respiratory failure, lung infection, anastomotic leakage (AL), or surgical site infection).

The determination of postoperative complications was based on the medical record system. These complications can be listed as follows; respiratory failure (postoperative arterial blood gas analysis PaO_2_ < 60 mmHg); lung infection, with clinical symptoms of cough, productive cough, fever or chest tightness, leukocyte count > 10.0 × 10^9^/L or < 4.0 × 10^9^/L, and purulent secretions, and postoperative imaging of new or progressive development, persistent pulmonary invasive shadows, and consolidation; anastomotic leakage (full thickness GI defect involving the esophagus, anastomosis, staple line, or conduit irrespective of presentation or method of identification); and surgical site infection (after the operation, the surgical incision exhibited an inflammatory reaction, pus, or wound infection requiring an opening wound or the use of antibiotics).

### Statistical analysis

Continuous variables with a normal distribution are expressed as the mean ± standard deviation (SD), continuous variables with a skewed distribution are expressed as the median (Q1, Q3), and categorical variables are expressed as a frequency or percentage. We used χ^2^ tests (categorical variables), Student’s t tests (normal distribution), or Mann–Whitney U tests (skewed distribution) to evaluate differences between the anesthesia (GA and E-GA) groups. To confirm the association between anesthesia type and PLOS, we conducted a sensitivity analysis using propensity score matching because anesthesia types differed significantly across baseline characteristics [17]. The rationale and methods of using propensity score matching in the context of cohort studies have been previously described [18–20]. We considered a confounder to be well balanced if the standardized difference was less than 0.1. Patients in the E-GA group were matched with those in the GA group at a ratio of 1:1 using greedy matching with a caliper of 0.01.

Data analysis can be summarized into two steps. Step 1: Selection bias was avoided by using propensity score matching; subsequently, univariate and multivariate linear regression were used to explore the relationship between anesthesia type and PLOS (days) (Table 2). Next, we used univariate and multivariate binary logistic regression models to examine the association between anesthesia type and prolonged PLOS (> 21 days) with three different models (Table 3). Variables with a P < 0.1 in univariate analysis were entered into the multivariate logistic regression model. Step 2: Subgroup analyses were performed using a stratified linear regression model [16]. For continuous variables, we first converted the variables to categorical variables according to the clinical cutoff point or tertile and then performed an interaction test. The likelihood ratio test followed tests for the effect of subgroup indicators. To ensure the robustness of the data analysis, we performed a sensitivity analysis [21]. All analyses were performed with the statistical software packages R (http://www.R-project.org, The R Foundation) and EmpowerStats (http://www.empowerstats.com, X&Y Solutions, Inc, Boston, MA). P values less than 0.05 (two-tailed) were considered statistically significant.

## Results

### Study population

The study initially involved a total of 680 participants. Participants’ entry time and selection deadline were 2010-1-1 and 2020-12-30, respectively. The inclusion criteria were patients undergoing radical esophageal malignant tumor resection while receiving GE or E-GA from January 01, 2010 to December 31, 2020. Exclusion criteria were (1) an unplanned second surgery (*n* = 8), (2) combined operation at other sites than the esophagus (*n* = 10), (3) automatic discharge or postoperative death (*n* = 5), (4) canceled operations (*n* = 4), (5) postoperative pathological results showing nonesophageal cancer (*n* = 4), and (6) missing data (*n* = 2). The final number of cases was 647. The mean age of the 647 patients selected was 61.01 ± 8.16 years old; of these patients, 77.43% were men, 185 underwent GA, and the remaining 462 underwent E-GA.

### Baseline characteristics of selected participants

The baseline characteristics of the selected participants are shown in Table 1. Before propensity score matching, the confounders were unevenly distributed between the GA and E-GA groups. The average age of the E-GA group (60.51 ± 8.11 years old) was lower than that of the GA group (62.44 ± 8.14 years old) (*P* < 0.05). The following confounders exhibited higher rates in the GA group than in the E-GA group: preoperative chemotherapy, hypertension, heart disease, Scr levels, ASA III, single lumen intubation, CIIV, MIE, operation time, vasoactive drug use, and postoperative admission to the ICU (*P* < 0.05). Participants in the E-GA group had higher values for albumin and blood loss. They experienced more double-lumen intubation, TIVA, OE, and surgical site infections than the GA group (*P* < 0.05). For unbiased comparisons, propensity score matching was performed to minimize intergroup differences among some cofounders. After propensity score matching, 137 patients in the E-GA group were successfully matched with 137 patients in the GA group (*P* > 0.05), and the confounders were uniformly distributed between the two groups.

**Table 1:** Comparison of variables in the GA and EGA groups before and after propensity score matching

Variable	Total	Before matching			After matching			
		GA group	E-GA group	*P* value	GA group	E-GA group	*P* value	Standardized diff.
N	647	185	462		137	137		
Age (years)	61.06 ± 8.16	62.44 ± 8.14	60.51 ± 8.11	0.006	61.89 ± 8.24	61.93 ± 7.77	0.964	0.0055
Male	501 (77.43%)	140 (75.68%)	361 (78.14%)	0.498	106 (77.37%)	101 (73.72%)	0.482	0.0850
History of smoking	342 (52.86%)	98 (52.97%)	244 (52.81%)	0.971	73 (53.28%)	76 (55.47%)	0.716	0.0440
Preoperative chemotherapy	142 (21.95%)	55 (29.73%)	87 (18.83%)	0.002	38 (27.74%)	38 (27.74%)	1.000	0.0000
Coexisting disease								
Hypertension	102 (15.77%)	41 (22.16%)	61 (13.20%)	0.005	25 (18.25%)	26 (18.98%)	1.0000	0.0188
DM	47 (7.26%)	16 (8.65%)	31 (6.71%)	0.391	11 (8.03%)	14 (10.22%)	0.6748	0.0761
Heart disease	67 (10.36%)	28 (15.14%)	39 (8.44%)	0.012	20 (14.60%)	20 (14.60%)	1.000	0.0000
Lung disease	150 (23.18%)	45 (24.32%)	105 (22.73%)	0.664	33 (24.09%)	35 (25.55%)	0.780	0.0338
Laboratory examination results								
Preoperative anemia	237 (36.63%)	72 (38.92%)	165 (35.71%)	0.445	51 (37.23%)	50 (36.50%)	0.900	0.0151
PLT (10^9^/L)	233.61 ± 76.60	228.64 ± 72.96	235.60 ± 78.00	0.297	232.38 ± 77.52	229.96 ± 76.89	0.795	0.0314
Albumin (g/L)	39.63 ± 4.24	39.11 ± 5.11	39.84 ± 3.82	0.049	39.02 ± 4.97	39.38 ± 3.57	0.493	0.0830
Scr (µmol/L)	90.51 ± 23.12	94.69 ± 26.21	88.84 ± 21.57	0.004	92.62 ± 15.7)	94.53 ± 23.96	0.435	0.0945
AST (mmol/L)	23.53 ± 10.93	24.49 ± 10.28	23.15 ± 11.17	0.161	24.63 ± 10.55	23.77 ± 10.80	0.506	0.0805
ALT (mmol/L)	17.00 (13.00–24.00)	17.00 (12.02-25.00)	17.00 (13.00–24.00)	0.731	17.00 (12.02-26.00)	17.00 (14.00–24.00)	0.920	0.0732
Intraoperative variables								
ASA				0.005			0.1756	0.1878
I/II	594 (91.81%)	161 (87.03%)	433 (93.72%)		118 (86.13%)	126 (91.97%)		
III	53 (8.19%)	24 (12.97%)	29 (6.28%)		19 (13.87%)	11 (8.03%)		
Endotracheal tube type				< 0.001			0.803	0.0302
Single lumen	254 (39.26%)	133 (71.89%)	121 (26.19%)		87 (63.50%)	85 (62.04%)		
Double lumen	393 (60.74%)	52 (28.11%)	341 (73.81%)		50 (36.50%)	52 (37.96%)		
Continuous anesthesia				< 0.001			1.000	0.0000
TIVA	587 (90.73%)	148 (80.00%)	439 (95.02%)		122 (89.05%)	122 (89.05%)		
CIIA	60 (9.27%)	37 (20.00%)	23 (4.98%)		15 (10.95%)	15 (10.95%)		
Operation type				< 0.001			0.787	0.0326
OE	347 (53.63%)	40 (21.62%)	307 (66.45%)		39 (28.47%)	37 (27.01%)		
MIE	300 (46.37%)	145 (78.38%)	155 (33.55%)		98 (71.53%)	100 (72.99%)		
Blood loss (ml)	200.00 (100.00-250.00)	100.00 (100.00-200.00)	200.00 (100.00-250.00)	0.004	100.00 (100.00-200.00)	100.00 (100.00-200.00)	0.746	0.0565
Operation time (min)	239.13 ± 56.72	259.61 ± 55.09	230.93 ± 55.32	< 0.001	256.43 ± 50.17	249.82 ± 46.57	0.260	0.1365
Vasoactive drug use	341 (52.70%)	109 (58.92%)	232 (50.22%)	0.045	82 (59.85%)	81 (59.12%)	0.902	0.0149
Blood transfusion	127 (19.63%)	42 (22.70%)	85 (18.40%)	0.213	26 (18.98%)	30 (21.90%)	0.549	0.0725
Postoperative ICU admission	42 (6.49%)	18 (9.73%)	24 (5.19%)	0.034	10 (7.30%)	12 (8.76%)	0.657	0.0537
Postoperative complications								
Respiratory failure	24 (3.71%)	9 (4.86%)	15 (3.25%)	0.325	6 (4.38%)	8 (5.84%)	0.583	0.0663
Lung infection	212 (32.77%)	58 (31.35%)	154 (33.33%)	0.627	41 (29.93%)	48 (35.04%)	0.367	0.1093
Anastomotic leakage	42 (6.49%)	11 (5.95%)	31 (6.71%)	0.722	8 (5.84%)	10 (7.30%)	0.626	0.0590
Surgical site infection	37 (5.72%)	5 (2.70%)	32 (6.93%)	0.037	4 (2.92%)	4 (2.92%)	1.000	0.0000
PLOS (days)	19.85 ± 12.60 16.00 (14.00–21.00)	20.01 ± 14.90 15.00 (14.00–20.00)	19.79 ± 11.57 16.00 (14.00–21.00)	0.094	18.09 ± 9.71 15.00 (14.00–18.00)	19.39 ± 10.75 16.00 (14.00–22.00)	0.145	0.1276

Data are expressed as the mean ± SD, median (Q1-Q3), or N (%)

*Abbreviations: GA *general anesthesia, *E-GA *combined epidural-general anesthesia, *DM *diabetes mellitus, *PLT *platelet, *AST *aspartate transaminase, *ALT *alanine transaminase, *Scr *serum creatinine, *ASA *American Society of Anesthesiologist Physical Status, *TIVA *total intravenous anesthesia, *CIIA *combined intravenous and inhalation anesthesia, *OE *open esophagectomy, *MIE *minimally invasive esophagectomy, *ICU *intensive care unit, *PLOS *postoperative length of stay

### Univariate and multivariate analyses

The results of the univariate analyses (after propensity score matching) are presented in Table 2. Variables with a *P* value < 0.1 in the univariate analysis were entered into a multivariate logistic regression model to identify risk factors for PLOS. Neither univariate nor multivariate analyses showed an association between anesthesia type and PLOS. The multivariate regression showed that lung infection (β = 3.35, 95% CI: 1.54–5.52, *P* = 0.006), anastomotic leakage (β = 25.73, 95% CI: 22.11–29.34, *P* < 0.001) and surgical site infection (β = 9.39, 95% CI: 4.10-14.68, *P* = 0.006) were significant risk factors for PLOS.

**Table 2:** Univariate and multivariate analyses of factors associated with PLOS (days)

	Univariate		Multivariate	
	β (95% CI)	*P* value	β (95% CI)	*P* value
Age (years)	0.11 (-0.04, 0.26)	0.1446		
Male	-0.36 (-3.19, 2.46)	0.8016		
History of smoking	-2.55 (-4.97, -0.13)	0.0399	-1.77 (-3.58, 0.04)	0.0561
Preoperative chemotherapy	-1.10 (-3.81, 1.61)	0.4280		
Coexisting disease				
Hypertension	1.14 (-1.98, 4.26)	0.4754		
DM	0.68 (-3.54, 4.90)	0.7519		
Heart disease	-0.55 (-3.99, 2.90)	0.7563		
Lung disease	1.89 (-0.91, 4.69)	0.1877		
Laboratory examination results				
PLT (10^9^/L)	0.00 (-0.01, 0.02)	0.6467		
Albumin (g/L)	0.09 (-0.20, 0.37)	0.5481		
Scr (µmol/L)	-0.01 (-0.07, 0.05)	0.6390		
AST (mmol/L)	-0.03 (-0.15, 0.08	0.2870		
ALT (mmol/L)	-0.04 (-0.12, 0.04)	0.2870		
Preoperative anemia	-1.72 (-4.23, 0.79)	0.1799		
Intraoperative variables				
ASA				
I/II	Reference			
III	-1.17 (-5.06, 2.72))	0.5563		
Anesthesia type				
GA	Reference		Reference	
E-GA	1.31 (-1.12, 3.73)	0.2920	0.76 (-1.01, 2.53)	0.4016
Endotracheal tube type				
Single lumen	Reference			
Double lumen	0.83 (-1.68, 3.35)	0.5155		
Continuous anesthesia				
TIVA	Reference			
CIIA	-1.02 (-4.91, 2.87)	0.6080		
Operation type				
OE	Reference			
MIE	-0.27 (-2.98, 2.45	0.8470		
Blood loss (ml)	0.00 (-0.01, 0.01)	0.5595		
Operation time (min)	0.02 (-0.01, 0.04)	0.1809		
Vasoactive drug use	-0.48 (-2.96, 1.99)	0.7035		
Blood transfusion	1.02 (-1.99, 4.03)	0.5067		
Postoperative ICU admission	6.31 (1.90, 10.72)	0.0054	2.99 (-1.83, 7.80)	0.2251
Postoperative complications				
Respiratory failure	5.24 (-0.24, 10.73)	0.0621	-0.76 (-6.61, 5.09)	0.7983
Lung infection	4.88 (2.35, 7.41)	0.0002	3.53 (1.54, 5.52)	0.0006
Anastomotic leakage	26.62 (22.87, 30.37)	< 0.0001	25.73 (22.11, 29.34)	< 0.0001
Surgical site infection	7.86 (0.71, 15.02)	0.0322	9.39 (4.10, 14.68)	0.0006

*Abbreviations: CI *confidence interval, *GA *general anesthesia, *E-GA *combined epidural-general anesthesia, *DM *diabetes mellitus, *PLT *platelet, *AST *aspartate transaminase, *ALT *alanine transaminase, *Scr *serum creatinine, *ASA *American Society of Anesthesiologist Physical Status, *TIVA *total intravenous anesthesia, *CIIA *combined intravenous and inhalation anesthesia, *OE *open esophagectomy, *MIE *minimally invasive esophagectomy, *ICU *intensive care unit, *PLOS *postoperative length of stay

In addition, we constructed three models to analyze the independent effects of the two types of anesthesia (GA and E-GA) on prolonged PLOS (> 21 days) after propensity score matching. Variables with *P* < 0.1 in the univariate analysis (Table 2) were entered into multivariate logistic regression model (Model II). The odds ratios (ORs) of prolonged PLOS and 95% confidence intervals (CIs) are listed in Table 3

As shown in Table 3, Model II showed that prolonged PLOS was 60% higher with E-GA than with GA (OR = 1.60, 95% CI 0.80–3.23, *P* = 0.1841)

**Table 3:** Relationship between the anesthesia type and prolonged PLOS (days)

Outcome	Prolonged PLOS (days) OR (95% CI) *P* value		
**Anesthesia type**	**Crude model**	**Model I**	**Model II**
GA	Reference	Reference	Reference
E-GA	1.54 (0.86, 2.74) 0.1456	1.54 (0.86, 2.78) 0.1473	1.60 (0.80, 3.23) 0.1841

*Abbreviations: PLOS *postoperative length of stay, *OR *odds ratio, *CI *confidence interval, *GA *general anesthesia, *E-GA *combined epidural-general anesthesia

Model I adjusted for age and sex

Model II adjusted for history of smoking, postoperative ICU admission, respiratory failure, lung infection, surgical site infection, and anastomotic leakage

### Subgroup analysis

As stratified variables, we selected categorical variables (sex, history of smoking, hypertension, DM, heart disease, lung disease, preoperative anemia, preoperative chemotherapy, ASA, endotracheal tube type, continuous anesthesia, operation type, vasoactive drug use, blood transfusion, postoperative ICU admission, respiratory failure, lung infection, anastomotic leakage, and surgical site infection) and continuous variables (age, PLT, AST, ALT, Scr, operation time, and blood loss) that were transformed into categorical variables. We then observed the differences in effect size for these variables (Table 4).

We found significantly different interactions for blood transfusion (*P* = 0.0346), operation type (*P* = 0.0346) and vasoactive drug use (*P* = 0.002). The remaining variables showed no significant differences.

**Table 4:** Effect size of anesthesia type on PLOS (days) in prespecified and exploratory subgroups

Anesthesia type	**PLOS (days)**		
	**N**	**β (95% CI)**	**P for interaction**
Age (years)			0.0897
≤60	116	-1.04 (-4.65, 2.57)	
>60	158	3.17 (-0.07, 6.41)	
Sex			0.5597
Female	67	0.03 (-3.53, 3.59)	
Male	207	1.70 (-1.30, 4.71)	
History of smoking			0.7850
No	125	1.00 (-3.18, 5.18)	
Yes	149	1.67 (-1.06, 4.39)	
Preoperative chemotherapy			0.8566
No	198	1.44 (-1.68, 4.57)	
Yes	76	0.95 (-2.25, 4.14)	
Hypertension			0.3587
No	223	0.76 (-2.03, 3.55)	
Yes	51	3.66 (-0.96, 8.28)	
DM			0.8682
No	249	1.36 (-1.25, 3.97)	
Yes	25	0.64 (-5.32, 6.61)	
Heart disease			0.0965
No	234	0.46 (-2.27, 3.19)	
Yes	40	6.25 (1.82, 10.68)	
Lung disease			0.5707
No	206	0.88 (-1.71, 3.47)	
Yes	68	2.49 (-3.37, 8.35)	
PLT (10^9^/L)			/
< 100	3	/	
≥100	271	1.40 (-1.04, 3.85)	
Hypoproteinemia			/
No	270	1.42 (-1.04, 3.88)	
Yes	4	/	
Scr (µmol/L) group			/
≤ 133	268	1.35 (-1.12, 3.83)	
> 133	6	/	
AST (mmol/L)group			0.5435
≤ 40	255	1.34 (-1.24, 3.92)	
> 40	19	1.09 (-3.65, 5.83)	
ALT (mmol/L) group			0.5750
≤ 40	248	1.06 (-1.58, 3.71)	
> 40	26	3.62 (-0.13, 7.36)	
Preoperative anemia			0.4957
No	173	0.65 (-2.87, 4.18)	
Yes	101	2.39 (-0.22, 4.99)	
ASA			/
I	13	6.77 (-3.79, 17.34)	
II	231	0.76 (-2.03, 3.54)	
III	30	2.77 (-1.01, 6.55)	
Endotracheal tube type			0.0680
Single lumen	172	-0.43 (-3.19, 2.33)	
Double lumen	102	4.21 (-0.33, 8.75)	
Continuous anesthesia			0.5115
TIVA	244	1.59 (-1.08, 4.26)	
CIIA	30	-1.00 (-5.38, 3.38)	
Operation type			0.0346
OE	76	5.50 (1.06, 9.94)	
MIE	198	-0.30 (-3.17, 2.58)	
Blood loss (ml)			/
≤ 400	267	1.28 (-1.21, 3.77)	
>400	7	/	
Operation time (min)			0.5442
≤ 280	212	1.81 (-0.37, 3.98)	
> 280	62	0.02 (-7.77, 7.82)	
Vasoactive drug use			0.0002
No	111	6.72 (2.64, 10.80)	
Yes	163	-2.39 (-5.25, 0.48)	
Blood transfusion			0.0346
No	218	1.08 (-1.81, 3.98)	
Yes	56	2.04 (-1.70, 5.78)	
Postoperative ICU admission			0.5396
No	252	1.00 (-1.38, 3.37	
Yes	22	3.75 (-8.90, 16.40)	
Respiratory failure			/
No	260	1.49 (-0.97, 3.95)	
Yes	14	-3.71 (-16.45, 9.03)	
Lung infection			0.7764
No	185	0.82 (-0.88, 2.53)	
Yes	89	1.55 (-4.88, 7.99)	
Anastomotic leakage			/
No	256	1.12 (-0.19, 2.44)	
Yes	18	-2.05 (-24.46, 20.36)	
Surgical site infection			/
No	266	1.38 (-1.07, 3.84)	
Yes	8	/	

The following variables were excluded because ≥ 5 categories or < 20 observations in a category: PLT (10^9^/L), AST (mmol/L), Scr (µmol/L), hypoproteinemia, ASA, blood loss (ml), respiratory failure, anastomotic leakage, and surgical site infection

*Abbreviations: PLOS* postoperative length of stay, *CI *confidence interval, *DM *diabetes mellitus, *PLT *platelet, *AST* aspartate transaminase, *ALT *alanine transaminase, *Scr *serum creatinine, *ASA *American Society of Anesthesiologist Physical Status, *TIVA *total intravenous anesthesia, *CIIA *combined intravenous and inhalation anesthesia, *ICU *intensive care unit; hypoproteinemia, albumin < 30 (g/L); preoperative anemia, hemoglobin < 130 g/L in males or hemoglobin < 120 g/L in females, *OE* open esophagectomy, *MIE *minimally invasive esophagectomy

## Discussion

Combined epidural-general anesthesia may be advantageous for patients receiving chest and abdominal surgery because epidural anesthesia can effectively inhibit sympathetic overexcitability, reduce the physiological stress response caused by surgery, reduce the use of opioids, and promote early postoperative gastrointestinal function; however, epidural anesthesia significantly increases the risk of arterial hypotension, pruritus, urinary retention, and motor blockade [22–26]. In our study, we found that the type of anesthesia had no significant effect on PLOS in patients undergoing radical resection of esophageal malignant tumor. This finding is consistent with the findings of Tankard, who did not find significant differences in any outcomes between regional and general anesthesia versus general anesthesia alone [14]. One systematic review indicated that there is no evidence to support or refute the use of epidural anesthesia or analgesia to reduce rates of cancer recurrence after gastroesophageal cancer surgery [25]; additionally, there is no difference in morbidity or mortality between analgesic treatments among patients undergoing esophagectomy [27]. In contrast, other studies reached different conclusions. Anesthesia and surgery can be seen as causing stress, trauma, and illness [28]. All of these can potentially increase PLOS. One study reported that total MIE under E-GA was associated with a longer hospital stay, probably due to the increased risk of anastomotic leakage with MIE compared to open or hybrid esophagectomy, but not this type of anesthesia was not associated with the risk of complications and readmission [11]; moreover, combined epidural-general anesthesia has been found to reduce the neuroinflammatory response and incidence of POCD as well as to improve short-term quality of life in patients with esophageal cancer [13, 26]

Postoperative complications are independently associated with decreased survival due to cancer recurrence [30], and prevention of complications may improve survival [31]. Wang W et al. reported that thoracic epidural anesthesia did not affect the risk of AL occurrence after esophageal surgery for cancer [32]. Technical complications substantially negatively impact survival after esophagogastrectomy for cancer [33]. A systematic review of 16 observational studies with 12,359 surgical patients demonstrated that diabetes is a significant risk factor for AL in patients undergoing esophagectomy [34]. Van Kooten RT et al. showed that male sex and diabetes were prognostic factors for anastomotic leakage and major complications. The reasons for our analysis are as follows: the need for observation and treatment after the occurrence of AL and delayed healing is bound to prolong PLOS; additionally, surgical technique, DM, [34] nutrition prior to surgery, [35] and early postoperative oral feeding [36] are influencing factors of AL. Postoperative epidural pain control can significantly decrease the incidence of pulmonary morbidity because it avoids the use of respiratory depressant opioids and improves ventilation function that increases PaO_2_ and early mobilization [37]. Epidural analgesia and the avoidance of intraoperative blood transfusion are significantly associated with a reduced 90-day mortality related to postoperative pulmonary complications from OE [38]. A meta-analysis reported that combined anesthesia provides better analgesia and fewer cases of postoperative respiratory failure [39]. The two-lung ventilation approach resulted in better intraoperative respiratory function and reduced PLOS (*P* < 0.05), although there was no significant difference in rates of postoperative respiratory complications [40]. Lung infection is a common complication of this operation, and methods of reducing or even preventing infection merit exploration. One study highlighted the influence of minimally invasive surgery, postoperative pain management, early identification of complications and the usage of uniform definitions on rates of lung complications after esophagectomy [41].

The wound length and pain in OE were greater than those in MIE. The advantage of MIE were no need for rib fractures, the ambulation early after surgery, less intraoperative blood loss, and lower total complication rates compared with OE [42–44]. However, data on which operation type is better are inconsistent. In one study, the proportion of patients who experienced serious adverse events, all adverse events, and the median LOS were significantly lower in the laparoscopic group than in the OE group [45]. A systematic analysis including 24 studies found that almost all of the nonrandomized studies demonstrated either a significant reduction in LOS with MIE or no difference [46]. In contrast, a retrospective study with propensity score matching showed that MIE (*n* = 3,515) was comparable to conventional OE (*n* = 3,515) in terms of short-term, with thoracic esophageal cancer patients who underwent esophagectomy at 864 hospitals (total *n* = 9,584) in Japan [47]. To compare the superiority of OE and MIE in the future, an article [48] provided relevant guidance: the use of nonrandom studies, complete transparency and fairness in patient allocation, clear baseline characteristics, descriptions of the experience of operating surgeons and the medical institute and longer follow-up. Our findings need to be confirmed by future studies

Vasoactive drugs were administered at the discretion of the anesthesiologist without a standard protocol due to there is no widely accepted definition of intraoperative hypotension. Intraoperative hypotension is associated with increased 30-day operative mortality in Noncardiac Surgery [49, 50]. The use of vasoactive drugs has been shown to correlate with an improved outcome in adult patients having major abdominal surgery because reduce postoperative complications and hospital length of stay [51]. Our study found that the use of vasoactive drugs may shorten PLOS. The use of vasoactive drugs in esophagectomy has been a source of controversy between surgeons and anesthesiologists, as the gastric tip of the anastomosis is only perfused by the gastric epithelial artery, and using vasoactive drugs has the potential to cause adverse effects due to ischemia. The administration of a thoracic epidural bolus may decrease flux at the anastomotic end of the gastric tube [52]. Vasoconstriction induced by the use of norepinephrine may be effective in restoring hypotension, but at the same time, the effects of vasoconstriction are even more dangerous than the hypotension itself. Some have suggested using liquid therapy instead of vasoactive drugs [53]. A prospective study including 54 patients showed that systolic blood pressure < 90 mmHg for more than 5 min was not significantly associated with individual or composite outcomes of mortality, AL, or prolonged hospital stay (OR = 1.06, P = 0.16) [54]. A retrospective study did not observe evidence that the intraoperative use of perioperative vasopressors or total fluid administration was associated with increased odds of perioperative anastomotic leakage following open Ivor Lewis esophagectomy [55].

Our study has the following advantages: (1) relatively large sample size for a single center study compared to a similar previous study; (2) strict statistical adjustment to minimize the residual confounders that observational studies are susceptible to; (3) handling independent variables as both continuous variables and categorical variables, which can reduce the contingency in the data analysis and enhance the robustness of the results; and (4) the use of effect modifier factor analysis to better utilize the data to draw stable conclusions in different subgroups

However, our study also has some limitations: (1) retrospective analysis performed in a single institution, which limits generalizability; (2) no postoperative care or early surgical rehabilitation; (3) patient-controlled intravenous analgesia in the GA group and patient-controlled epidural analgesia in the E-GA group, without unification of the postoperative analgesia; (4) lack of severity classification of postoperative complications; and (5) lack of identification of contraindications for epidurals in the GA group

## Conclusion

Although there is no significant association between the type of anesthesia(GA or E-GA) and PLOS for patients undergoing radical esophageal malignant tumor resection, an association between PLOS and lung infection, anastomotic leakage, and surgical site infection was determined by multivariate regression analysis. A larger sample future study design may verify our results

## Data Availability

The datasets generated during and analyzed in the current study are not publicly available due to institutional restrictions but are available from the corresponding author on reasonable request. .

## Abbreviations

GA: general anesthesia
E-GA: combined epidural-general anesthesia
AL: anastomotic leakage
ALT: alanine transaminase
AST: aspartate transaminase
ASA: American Society of Anesthesiologist Physical Status
CI: confidence interval
CIIA: combined intravenous and inhalation anesthesia
DM: diabetes mellitus
EC: esophageal cancer
ICU: intensive care unit
MIE: minimally invasive esophagectomy
LOS: length of stay
OE: open esophagectomy
OR: odds ratio
PLOS: postoperative length of stay
PaO_2_: arterial partial pressure of oxygen
POCD: postoperative cognitive dysfunction
PLT: platelet
Scr: serum creatinine
SD: standard deviation
TIVA: total intravenous anesthesia
